# Integrated lipidomic and transcriptomic analyses reveal the mechanism of lipid biosynthesis and accumulation during seed development in sesame

**DOI:** 10.3389/fpls.2023.1211040

**Published:** 2023-06-22

**Authors:** Yujuan Zhang, Huihui Gong, Xinxiao Cui, Chunhua Gao, Nana Li, Yanyan Pu, Xiurong Zhang, Junsheng Zhao

**Affiliations:** ^1^Institute of Industrial Crops, Shandong Academy of Agricultural Sciences, Jinan, China; ^2^Institute of Crop Germplasm Resources, Shandong Academy of Agricultural Sciences, Jinan, China

**Keywords:** sesame, seed development, lipidome, transcriptome, fatty acid, lipid biosynthesis

## Abstract

Sesame is one of the most important oilseed crops and attracts significant attention because of its huge nutritional capacity. However, the molecular mechanisms underlying oil accumulation in sesame remains poorly understood. In this study, lipidomic and transcriptomic analyses in different stages of sesame seed (Luzhi No.1, seed oil content 56%) development were performed to gain insight into the regulatory mechanisms that govern differences in lipid composition, content, biosynthesis, and transport. In total, 481 lipids, including fatty acids (FAs, 38 species), triacylglycerol (TAG, 127 species), ceramide (33 species), phosphatidic acid (20 species), and diacylglycerol (17 species), were detected in developing sesame seed using gas and liquid chromatography-mass spectrometry. Most FAs and other lipids accumulated 21–33 days after flowering. RNA-sequence profiling in developing seed highlighted the enhanced expression of genes involved in the biosynthesis and transport of FAs, TAGs, and membrane lipids, which was similar to that seen during lipid accumulation. Through the differential expression analysis of genes involved in lipid biosynthesis and metabolism during seed development, several candidate genes were found to affect the oil content and FA composition of sesame seed, including *ACCase*, *FAD2*, *DGAT*, *G3PDH*, *PEPCase*, *WRI1* and *WRI1-like* genes. Our study reveals the patterns of lipid accumulation and biosynthesis-related gene expression and lays an important foundation for the further exploration of sesame seed lipid biosynthesis and accumulation.

## Introduction

Vegetable oils are not only an essential part of the human daily diet and play an important role in nutrition but are also important contributors to chemical feedstocks and renewable biofuels (Dyer et al., 2008; Lummiss et al., 2012). In nature, some plants can synthesize a large amount of oil in seed, pollen, some fleshy fruits, and other organs, and store them in the form of triacylglycerol (TAG), which mainly include oilseed crops (e.g., soybean, rapeseed, peanut, sesame, and sunflower), perennial woody oil plants (e.g., oil palm, coconut, oil-tea camellia, and walnut), and some fiber crops such as cotton, flax, and hemp, among others (Zhang et al., 2009). Recently, with the improvement in living standards and the development of the economy, the worldwide consumption of vegetable oils is increasing year by year and there is more demand for high-quality edible oils (Lu et al., 2011). Therefore, it would be of great scientific and practical significance to elucidate the molecular mechanism of the biosynthesis and regulation of oil in plants.

In seed, oil mostly occurs in the form of triacylglycerol (TAG) which is composed primarily of three fatty acids (FAs) esterified to a glycerol backbone and stored in cytosolic oil bodies or liposomes (Kelly et al., 2013b; Bansal et al., 2018). In recent years, the molecular mechanism of the biosynthesis and regulation of oil in plants have been studied intensively (Harwood, 1996; Bates et al., 2013; Li-Beisson et al., 2013; He and Ding, 2020; Yang et al., 2022). Lipid biosynthesis and accumulation is a complex process in plant cells. Lipids are formed by *de novo* synthesis in the plastid by elongation and desaturation of FAs; assembly of TAGs occurs in the endoplasmic reticulum (ER); and finally, the oil body forms in the seed (Harwood, 1996; Bates et al., 2013; He and Ding, 2020). Many genes and gene products involved in controlling oil biosynthesis, including enzymes, directly participate in FA and TAG biosynthetic pathways. In *Arabidopsis*, TAG biosynthesis and its regulation has been well characterized. Acyl-lipid metabolism requires 120 enzymatic reactions and more than 600 genes to encode proteins and regulatory factors (Li-Beisson et al., 2013). Based on the genes that encode the key enzymes that participate in lipid biosynthetic and metabolic pathways in *Arabidopsis*, many homologous genes have been cloned and validated their functions in oilseed crops, such as *ACS* (Ding et al., 2020), *SAD* (Knutzon et al., 1992), *LPAAT* (Bourgis et al., 1999), *DATG* (Chen et al., 2016), and *FAD* (Haun et al., 2014). In addition, several crucial transcription factors (TFs), have been identified and play important roles in the process of seed maturation and lipid accumulation. Examples include the LEC (Tan et al., 2011), WRI (Chen et al., 2020), MYB (Liu et al., 2014), bZIP (Song et al., 2013), DOF (Wang et al., 2007), as well as NFYA TF families (Kelly et al., 2013a). Moreover, several new genes that regulate lipid biosynthesis in oilseed crops have also been identified (Elhiti et al., 2012; Liu et al., 2012; Kelly et al., 2013a).

Sesame (*Sesamum indicum* L.) is an important oil and cash crop worldwide, and is grown widely in several countries in Asia and Africa such as China, India, and Sudan due to its high oil content and quality (Zhang et al., 2013; Qureshi et al., 2022). Their seed oil contains a huge amount of unsaturated FAs, such as linoleic acid, oleic acid, and alpha-linolenic acid, which determine their important nutritional and economic values (Wei et al., 2015; Melo et al., 2021). Previous research has found that sesame oil contains a stably high proportion of unsaturated FAs, ranging from 83% to 87% (Wei et al., 2015). Furthermore, as a rich source of natural antioxidants, such as sesamin and sesamol, sesame seed is an important source of oil, with high nutritional value, and the global demand for various sesame products is increasing (Figueiredo et al., 2017). Therefore, improving the oil content and quality of sesame seed is an important and ongoing objective for sesame breeding. *SiNST1* and *SiPPO*, as well as several other genes (including *SiACNA*, *SiDGAT2*, *SiFATA*, *SiFATB*, and *SiSAD*), detected as candidate genes that control oil content in sesame were found to be involved in lipid metabolism (Wei et al., 2015). Despite this, no specific function has been clarified for these genes, and our knowledge of the biosynthesis and regulation of oil in sesame is still limited. The correlation between changes in transcriptome and lipid accumulation during seed development in sesame has not been explored.

Here, to understand the lipid composition during sesame seed development and the regulatory mechanism that governs the process of lipid accumulation in sesame, both transcriptomic and lipidomic profiling of sesame seed at three different developmental stages were conducted. This study aimed to investigate the network and candidate genes highly associated with sesame seed oil synthesis and to provide a better theoretical basis for the molecular improvement and genetic breeding of sesame and other oilseed crops.

## Materials and methods

### Sample preparation

Sesame accession Luzhi No.1, which has a seed oil content of 56%, was used in this study. The sesame was grown in the experimental field of the Shandong Academy of Agricultural Sciences. Seeds from capsules at the middle part of the sesame plants were harvested at 9, 21, and 33 days (d) after flowering, representing early, middle, and late stages of Luzhi No.1 seed maturation, respectively. All seed samples with three biological replicates were flash-frozen in liquid nitrogen and stored at −80°C.

### Fatty acid determination

Determination of FA samples of Luzhi No.1 at 9, 21, and 33 d was performed by gas chromatography coupled with mass spectrometry (GC-MS) at Shanghai Applied Protein Technology Company, according to a method described previously (Zhu et al., 2019). In brief, seed samples (50 mg) were frozen and ground in liquid nitrogen. A solution of chloroform:methanol (3:2, v/v) was used to extract FAs. After ultrasonication for 30 min, 2 mL of 1% sulfuric acid in methanol were added to the supernatant. For methyl esterification, the mixture was incubated in an 80°C water bath for 30 min to achieve FA esterification. Then, 1 mL n-hexane and 5 mL water were added and vortex-mixed. The supernatant (500 µL) was spiked with an internal standard (25 µL of 500ppm methyl salicylate), mixed, and subjected to GS-MS using an Agilent Model 7890A/5975C GC-MS system with an Agilent DB-WAX capillary GC column (30 m × 0.25 mm × 0.25 µm).

Conditions for chromatography were as follows: the initial temperature was 50°C and was maintained for 3 min. The temperature was then increased to 220°C at 10°C/min, and maintained at 220°C for 20 min. The carrier gas was helium (1.0 mL/min). A quality-control sample was used for testing and evaluating the stability and repeatability of the system. The temperatures of the injection port and transmission line were 280°C and 250°C, respectively. The electron bombardment ionization source, SIM (Selected ion Monitor) scanning mode, and electron energy were 70 eV. To quantify medium- and long-chain FA, MSD ChemStation software was used to extract retention time and chromatographic peak area, and Supelco 37-component FAME (FA methyl ester) mix (Sigma-Aldrich) was used to construct a calibration curve for the concentration range of 0.5–1000 mg/L. FA content was expressed as µg g^−1^ fresh weight (FW). One-way ANOVA was used to compare data between multiple groups, followed by the Student *t*-test to compare data between two groups. *P*-values below 0.05 were deemed statistically significant.

### Lipid extraction and targeted lipidomic analysis

Total lipid was extracted from samples of Luzhi No.1 at 9, 21, and 33 d, which represents early, mid, and late phases of seed development in sesame, according to a method reported previously (Taguchi and Ishikawa, 2010). Lipid measurements and data analysis were performed by high-performance liquid chromatography-mass spectrometry (LC-MS), according to the Shanghai Applied Protein Technology Company (Shanghai, China) standard procedures (Zhu et al., 2019). A methyl tert-butyl ether method was used for sample preparation and lipid extraction. In brief, seed material (40 mg) was accurately weighed and spiked with appropriate amounts of internal lipid standards (SPLASH^®^ LIPIDOMIX MASS SPRC STANDARD, AVANTI,330707-1EA). After homogenizing with 200 uL distilled water, 240 uL pre-cooled methanol, and 800 uL methyl tert-butyl ether, a gentle stream of nitrogen was used to dry the total lipid extract. Quality control (QC) samples were prepared by pooling and mixing 10 uL of each sample. These were run at the beginning of the sample queue to condition the column, and every 10 injections thereafter to assess inconsistencies, which are particularly evident in large batch acquisitions in terms of retention time drifts and variation in ion intensity over time.

For LC-MS/MS, reverse phase chromatography was selected for LC separation using a CSH C18 column (1.7 μm, 2.1 mm × 100 mm, Waters). The lipid extracts were re-dissolved in 200 μL of 90%isopropanol/acetonitrile, centrifuged at 14,000× g for 15 min, and finally 3 μL of sample was injected. Solvent A was acetonitrile–water (6:4, vol/vol) containing 0.1% formic acid and 0.1 mM ammonium formate, and solvent B was acetonitrile–isopropanol (1:9, vol/vol) containing 0.1% formic acid and 0.1 mM ammonium formate. The gradient elution was programmed as follows: the initial mobile phase contained 30% solvent B and 70% solvent A at a flow rate of 300 μL/min. It was held for 2 min, and then solvent B was linearly increased to 100%, while solvent A was decreased to 0% from 2–25 min. This was followed by equilibration in 5% solvent B and 95% solvent A from 25–35 min. Mass spectra were acquired using a Q-Exactive Plus system in positive and negative modes, respectively. ESI (Electron Spray Ionization) parameters were optimized and preset for all measurements as follows: source temperature, 300°C; capillary temperature, 350°C; ion spray voltage, 3000 V, S-Lens RF Level was set at 50%. The MS1 scan range was 200-1800 m/z. The mass:charge ratios were determined for lipid molecules and fragments; 10 fragments (MS2 scan, HCD) were collected after each full scan. MS1 occurred at a resolution of 70 000 at m/z 200 and MS2 occurred at a resolution of 17 500 at m/z 200.

Lipidsearch (Thermo Fisher Scientific, USA) was used to extract and identify the lipid molecule and internal standard peaks. The main parameters were as follows: precursor tolerance: 5 ppm; product tolerance: 5 ppm; and product ion threshold: 5%.

### Data processing and statistical analysis

Lipid species were identified using LipidSearch software version 4.2 (Thermo Scientific™) to process the raw data and for peak alignment, retention time correction, and peak area. LipidSearch contains data on more than 30 lipid classes, with information on more than 1,700,000 ion fragments. In the data extracted from LipidSearch, the ion peak with > 50% missing values were removed, and the Perato scaling method was used for normalization (Want et al., 2010). The absolute amount of each lipid ion was calculated using a single-point calibration method, as described previously (Want et al., 2010). Lipid content was expressed as µg g-1 FW. One-way ANOVA was used to compare data between multiple groups, followed by Student’s *t*-test to compare data between two groups. *P*-values below 0.05 were deemed statistically significant.

### RNA extraction and transcriptomic analysis

Total RNA from fresh seed of Luzhi No.1 was extracted using an EASYspin Plus kit (Aidlab, Beijing, China), according to the manufacturer’s instructions. RNA quality was assessed using the Agilent Bioanalyzer. RNA-Seq libraries were prepared and sequenced on an Illumina Hiseq2500 platform at Shanghai Applied Protein Technology Company and subsequent data processing was performed as described previously (Zhu et al., 2019). Raw reads of fastq format were processed through in-house perl scripts and clean reads were obtained by removing those containing adapters, poly-Ns, and low-quality reads from raw data. The clean reads were mapped to the sesame reference genome v.1.0 (https://www.ncbi.nlm.nih.gov/genome/?term=sesamum) using HISAT2 (v2.0.5). Gene expression level for each sample was normalized by calculating fragments per kilobase of transcript per million fragments mapped (FPKM). The difference between transcriptome samples was performed using the DESeq2 and genes with adjusted *P*-value < 0.05 and |log2foldchange| >1 were assigned as differentially expressed. The KEGG pathway enrichment analysis was performed for differentially expressed genes (DEGs) using the clusterProfiler R package with a corrected *P*-value < 0.05 regarded as significantly enriched.

### qRT-PCR

The qRT-PCR was performed with ChamQTM SYBR qPCR Master Mix (Vazyme Biotech, Nanjing, China) on a LightCycler480 Real-Time PCR System, according to a method described previously (Zhang et al., 2019). The gene primers for qRT-PCR are listed in **Supplementary Table S1**. The sesame *actin7* gene was used as an endogenous control gene to normalize all target gene expression (Wei et al., 2015).

### Statistical analysis

For statistical analysis, all experiments were performed at least three times. Principal component analysis (PCA) and Pearson correlation were performed using the R statistical environment (version 3.5.1).

## Results

### Patterns of FA accumulation in developing sesame seed

**Figure 1:**
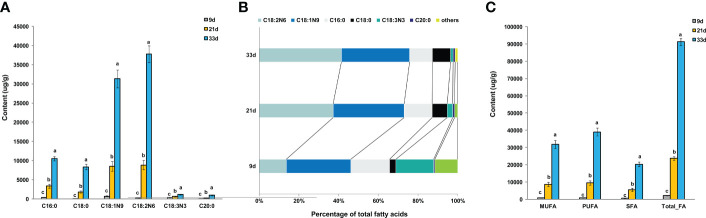
Analysis of FAs in developing sesame seed. **(A)** The levels of the most abundant FAs species. **(B)** Percentage composition of the main FA species at different developmental periods. **(C)** Saturated and unsaturated FA levels. n3, n6, and n9, the first double bonds in unsaturated FAs, occur at the 3, 6, and 9 positions of the methyl end of the carbon chain, respectively; FA, fatty acid; MUFA, monounsaturated fatty acids; PUFA, polyunsaturated fatty acids; SFA, saturated fatty acid. Different lowercase letters indicate a significant difference between the groups according to Student’s *t*-test (*P* < 0.05).

The oil content of the sesame seed at 9, 21, and 33 days after flowering was approximately 6%, 45%, and 56%, respectively. It is therefore crucial to understand the characteristics of FA accumulation and identify the correlations between the FAs during seed development in sesame. The changes in FA composition and level in developing seeds were analyzed by GC-MS. In total, 38 FA species, including medium- and long-chain FAs were identified. FA composition did not change significantly across developmental stages (**Table S2**). At the beginning of storage, all FA species showed lower levels, however, 16 and 26 FA species significantly increased at 21 and 33 d, respectively (**Table S2**). At both 21 and 33 d, palmitic acid (C16:0), stearic acid (C18:0), oleic acid (C18:1n9), and linoleic acid (C18:2n6) were the most abundant FAs, accounting for approximately 95% of the total FAs (**Figures 1A, B**). Phenomenally, linoleic acid (C18:2n6) and stearic acid (C18:0) were increased (percentage of total FAs) during seed development, while palmitic acid (C16:0) and linolenic acid C18:3n3 showed the opposite trend (**Figure 1B**). Although the content of oleic acid (C18:1n9) in 21 and 33 d continued to increase, its relative ratio of total FAs did not significantly change during the developmental period examined (**Figure 1B**). Moreover, it was observed that more than 72% of the total FAs, including monounsaturated FA (MUFA), polyunsaturated FA (PUFA), and saturated FA (SFA), accumulated strongly from 21 to 33 days after flowering, which suggested the critical period for rapid accumulation of FAs is in the 21–33 d period of storage (**Figure 1C**).

### Altered profiles of lipids across developmental stages of sesame seed

**Figure 2:**
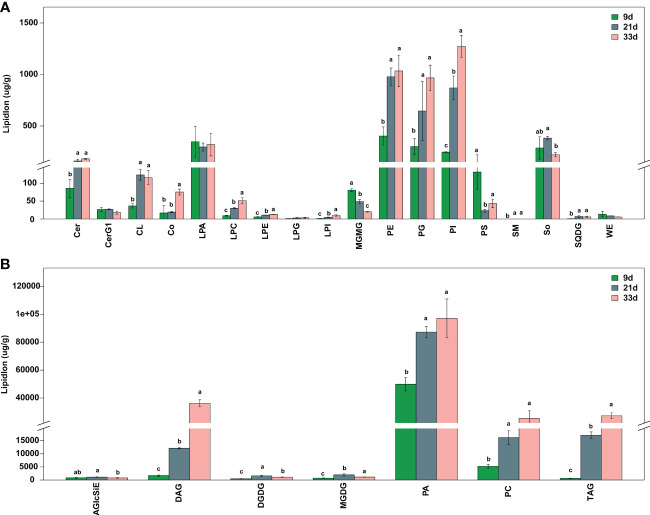
Total contents of lipid classes in seed at different developmental stages. **(A)** Low-abundance lipid species. **(B)** High-abundance lipid species. AGlcSiE, acylglcsitosterol ester; Cer, ceramide; CerG1, simple glc series; CL, cardiolipin; Co, coenzyme; DAG, diglyceride; DGDG, digalactosyldiacylglycerol; LPA, lysophosphatidic acid; LPC, lysophosphatidylcholine; LPE, lysophosphatidylethanolamine; LPG, lysophosphatidylglycerol; LPI, lysophosphatidylinositol; MGDG, monogalactosyldiacylglycerol; MGMG, monogalactosyldiacylglycerol; PA, phosphatidic acid; PC, phosphatidylcholine; PE, phosphatidylethanolamine; PG, phosphatidylglycerol; PI, phosphatidylinositol; PS, phosphatidylserine; SM, sphingomyelin; So, sphingosine; SQDG, sulfoquinovosyldiacylglycerol; TAG, triacylglycerol; WE, wax esters. Different lowercase letters indicate a significant difference between the groups according to Student’s *t*-test (*P* < 0.05).

To further understand the changes in other lipids during seed development, LC-MS was used to obtain lipidomic profiling of the sesame seed at 9, 21, and 33 d after flowering. The PCA of the lipidomic data showed that biological replicate samples were closely clustered together, indicating the experiment had good repeatability (**Figure S1**). The LC-MS analysis indicated that a total of 25 lipid classes containing 443 lipid species were detected in all samples of developing seed. Among them, 358 lipid species were differentially accumulated at 21 or 33 d, however, their composition was not changed from 9 to 33 d after flowering (**Table S3**). Compared to 9 d, quantification data reveal that the content of most lipid classes increased at 21 and 33 d, among which were triacylglycerol (TAG, 127 species), ceramide (Cer, 33 species), phosphatidic acid (PA, 20 species), diacylglycerol (DAG, 17 species), and so on (**Table S3**: **Figure 2**). In particular, the contents of total PA at 9, 21, and 33 d accounted for 81%, 62%, and 50% of the total lipids respectively, which were higher than other lipid classes (**Figure 2**).

**Figure 3:**
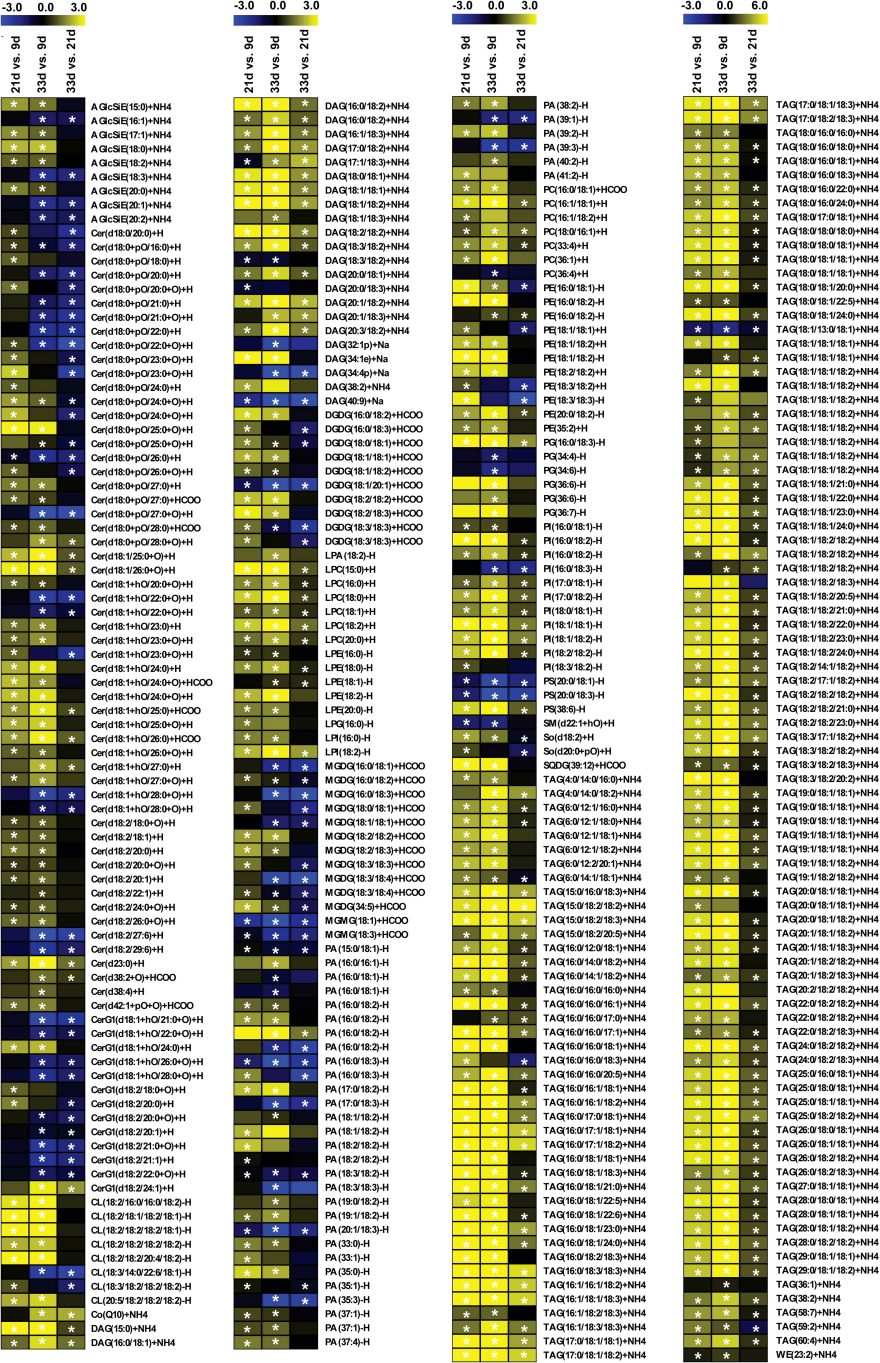
Heatmaps of content changes of lipids in developing seed. Yellow denotes increased lipid content and blue denotes decreased lipid content. AGlcSiE, acylglcsitosterol ester; Cer, ceramide; CerG1, simple glc series; CL, cardiolipin; Co, coenzyme; DAG, diglyceride; DGDG, digalactosyldiacylglycerol; LPA, lysophosphatidic acid; LPC, lysophosphatidylcholine; LPE, lysophosphatidylethanolamine; LPG, lysophosphatidylglycerol; LPI, lysophosphatidylinositol; MGDG, monogalactosyldiacylglycerol; MGMG, monogalactosyldiacylglycerol; PA, phosphatidic acid; PC, phosphatidyl choline; PE, phosphatidylethanolamine; PG, phosphatidylglycerol; PI, phosphatidylinositol; PS, phosphatidylserine; SM, sphingomyelin; So, sphingosine; SQDG, sulfoquinovosyldiacylglycerol; TAG, triacylglycerol; WE, wax esters. Heat maps were constructed based on the log_2_FoldChange levels for each lipid between 21, 33, and 9 d. Asterisks (*) indicate that mean values are significantly different between 9, 21, and 33 d, according to Student’s *t*-test (P < 0.05).

The accumulation of lipid species in the developing sesame seed was complicated, and the significant changes in pairwise comparisons between three developmental stages are shown in **Figure 3**. The accumulation of most phospholipids rapidly increased at 21 d; however, some phospholipids decreased or did not change at 33 d (**Figure 3**; **Table S3**). Among 35 differentially accumulated PAs, 3 PA species showed higher levels (>10000 µg g^−1^) in at least one developmental period of sesame seed, including 16:0/18:1, 16:0/18:2, and 18:2/18:2 PAs (**Figure 3**; **Table S3**). The contents of 16:0/18:1, 16:1/18:1, 18:0/16:1, 33:4 and 36:1 phosphatidylcholines (PCs) increased significantly at 21 and 33 d (**Figure 3**). In particular, 16:0/18:1 PC showed the highest values in all samples and was a major contributor to the rise in total PCs (**Table S3**). Compared to PAs and PCs, the levels of phosphatidylethanolamines (PEs), phosphatidylglycerols (PGs), phosphatidylinositols (PIs), and phosphatidylserines (PSs) were lower in developing seed (**Table S3**). For the DAGs and TAGs, the contents of most species steadily increased across developmental stages (**Figure 3**; **Table S3**). A total of 24 DAGs and 132 TAGs species differentially accumulated during seed development. In particular, the accumulation of 18:2/18:2, 18:1/18:2 DAG, and 18:1/18:2/18:2, 18:2/18:2/18:2 TAG was prominent at 21 and 33 d, which showed higher levels in developing seed (**Figure 3**; **Table S3**). Although 56 Cer, 9 AcylGlcSitosterol esters (AGlcSiEs) and 10 and 13 major galactolipid species, digalactosyldiacylglycerols (DGDGs) and monogalactosyldiacylglycerol (MGMG), respectively, showed significant changes, their levels were lower during sesame seed development (**Figure 3**; **Table S3**).

### Transcriptome analysis of developing sesame seed

**Figure 4:**
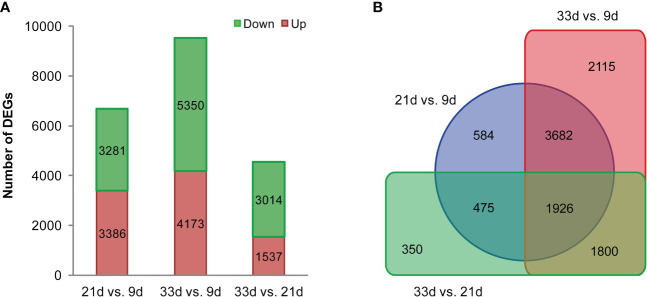
Numbers of differentially expressed genes (DEGs). **(A)** Pairwise comparisons for each stage of development. **(B)** Venn diagram of DEGs during seed development in sesame.

During sesame seed development, a transcriptome profile was examined for developing seed at 9, 21, and 33 d after flowering with three biological replicates. For each library, raw reads, clean reads, clean bases, Q20 (%), Q30 (%), GC (%), and the mapped percentage were recorded in **Table S4**. The Pearson correlation analysis showed that all libraries from the biological replicates were highly related, therefore, the quality of the samples in this sequencing was high enough for gene expression analysis (**Figure S2**). To investigate the dynamic changes in sesame transcriptome during seed development, pairwise comparisons between three developmental stages were performed. The gene expression levels were assessed by FPKM. A total of 10932 DEGs were identified between three developmental stages, and 6667, 9523, and 4551 DEGs were found at 21 vs. 9 d, 33 vs. 9 d, and 33 vs. 21 d, respectively (**Figure 4**). It was notable that the comparison of 33 vs. 9 d had the largest number of DEGs, with 4173 and 5350 genes exhibiting significantly up or down-regulated expression, respectively (**Figure 4A**). Interestingly, out of all the DEGs, 1926 DEGs were significantly differentially expressed in the two pairwise comparisons between the three developmental stages. KEGG pathway enrichment analysis showed that DEGs were mainly enriched in lipid biosynthesis and metabolic, carbohydrate, and energy metabolism pathways (**Table S5**), in particular, in “fatty acid biosynthesis (sind00061)”, “fatty acid elongation (sind00062)”, “fatty acid metabolism (sind01212)”, “biosynthesis of unsaturated fatty acids (sind01040)”, “alpha-linolenic acid metabolism (sind00592)”, “glycerolipid metabolism (sind00561)”, “phosphatidylinositol signaling system (sind04070)”, “glycosphingolipid biosynthesis (sind00603)”, and “glycolysis/gluconeogenesis (sind00010)”.

Analyses by qRT-PCR were performed on 10 selected DEGs related to lipid biosynthesis and metabolism. In all these genes, there was a very good correlation between RNA-seq and qRT-PCR data, indicating that the RNA-seq data are reliable (R^2 =^ 0.83, **Figure S3**).

### Expression pattern of genes related to FA biosynthesis and TAG assembly

**Figure 5:**
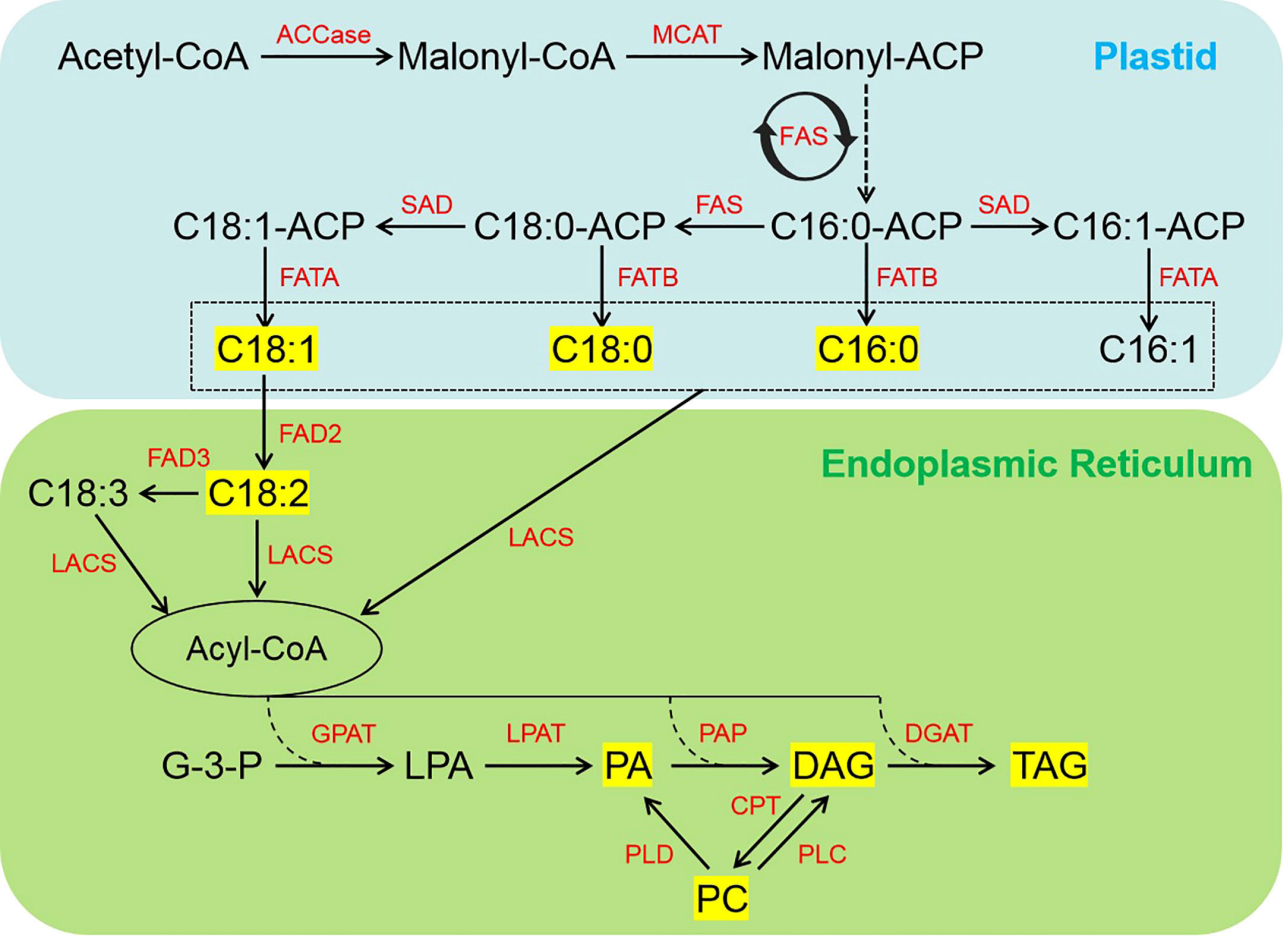
Simplified schematic of FA biosynthesis and TAG assembly pathways during seed development in sesame. The yellow box indicates lipids with content significantly higher in developing sesame seed. ACCase, acetyl-CoA carboxylase; CPT, diacylglycerol cholinephosphotransferase; DAG, diacylglycerol; DGAT, diacylglycerol acyltransferase; FA, fatty acid; FAS, fatty acid synthase; FATA, acyl-ACP thioesterase A; FATB, acyl-ACP thioesterase B; FAD2, fatty acid desaturase 2; FAD3, fatty acid desaturase 3; G-3-P, glycerol-3-phosphate; GPAT, glycerol-3-phosphate acyltransferase; LACS, long-chain acyl-CoA synthetase; LPA, lysophosphatidic acid; LPAT, lysophosphatidic acid acyltransferase; MCAT, malonyl CoA-ACP transacylase; PA, phosphatidic acid; PAP, phosphatidic acid phosphatase; PC, phosphatidylcholine; PLC, phospholipase C; PLD, phospholipase D; SAD, stearoyl-ACP desaturase; TAG, triacylglycerol.

Schematic and expression patterns of DEGs involved in FA biosynthesis and TAG assembly in sesame seed at different developmental stages are shown in **Figure 5** and **Table S6**, respectively. Acetyl-CoA carboxylase (ACCase, EC:6.4.1.2), the first key rate-limiting enzyme in FA biosynthesis, catalyzes the formation of malonyl-CoA from acetyl-CoA in plastids (**Figure 5**). In this study, nine putative members of *ACCase* gene showed the highest expression level at 21 d of seed development (**Table S6**). Note that another *ACCase* gene, LOC105161564, was highly expressed continuously during seed development (**Table S6**). The soluble stearoyl-ACP desaturase (SAD, EC:1.14.19.2) is membrane-bound and catalyzes the key step in unsaturated fat biosynthesis, and we found that the expression of five *SADs* was dramatically up-regulated in 21 and 33 d samples (**Figure 5**; **Table S6**). The FA desaturase (FAD, EC:1.14.19.6/1.14.19.22), catalyzes the introduction of double bonds at specific positions of FAs and thus the ratio of saturated to unsaturated FAs in seed (**Figure 5**). Expression of *FAD2* (LOC105159686) was significantly down-regulated at 33 d while two *FAD3* members (LOC105160895, LOC105167042) were significantly down-regulated at 21 and 33 d of seed development, which is consistent with the C18:2 and C18:3 accumulation patterns in developing sesame seed (**Table S6**; **Figure 1B**). These findings revealed that the regulation of *SAD* and *FAD2* levels during the middle and later stages of seed development may have a crucial role in the accumulation of oleic acid (C18:1n9) and linoleic acid (C18:2n6). Generally, the majority of the genes involved in FA biosynthesis showed higher levels of expression at 21 and 33 d after flowering, which is consistent with the changes in the accumulation of non-esterified FAs (**Table S6**). In addition, other important genes encoding enzymes involved in TAG biosynthesis, such as glycerol-3-phosphate acyltransferase (GPAT, EC:2.3.1.15), lysophospholipid acyltransferase (LPAT, EC:2.3.1.51), phosphatidate phosphatase (PAP, EC:3.1.3.81/3.1.3.4), and diacylglycerol acyltransferase (DGAT, EC:2.3.1.15), have also been detected in this study (**Figure 5**; **Table S6**). Higher transcript levels of *GPATs*, *PAPs*, *DGATs*, and two *LPAT* members were observed at 33 d after flowering (**Table S6**) and matched well with the change in the content of some DAG and TAG species (**Figure 3**).

### Expression pattern of genes related to carbohydrate metabolism

Glycerol-3-phosphate (G-3-P) and acetyl-CoA produced by glycolysis/gluconeogenesis are the raw materials for FA biosynthesis and TAG assembly, indicating that carbohydrate metabolism may have important implications for the improvement of oil content in plants. According to KEGG pathway enrichment analysis, several DEGs involved in carbon metabolism (sind01200), glycolysis/gluconeogenesis (sind00010), galactose metabolism (sind00052), starch and sucrose metabolism (sind00500), and pyruvate metabolism (sind00620) were identified during sesame seed development (**Table S5**). In plants, glycerol-3-phosphate dehydrogenase (G3PDH, EC:1.1.1.8) catalyzes dihydroxyacetone phosphate to form G-3-P. In general, the expression of four *G3PDH* members was distinctly up-regulated at 21 and 33 d, with LOC105176298 showing the highest transcript level (**Table S7**). Seven genes with generally higher transcript abundance in seed 21 d after flowering encode pyruvate dehydrogenases (PDHCs), which catalyze the synthesis of acetyl-CoA from pyruvate (**Table S7**). Notably, four genes encoding phosphoenolpyruvate carboxylase (PEPCase, EC:4.1.1.31) were found to be differentially expressed during sesame seed development, with *PEPCase 2* and *PEPCase 4* significantly down-regulated while *PEPCase* and *PEPCase 1-like* were significantly up-regulated (**Table S7**). Pyruvate kinase (PK, EC:2.7.1.40) is a key glycolytic regulatory enzyme, converting phosphoenol-pyruvate into pyruvate. The expression of a gene (LOC105161300) encoding PK was found to be increased persistently during sesame seed development, whereas four other *PK* genes were significantly up-regulated only in 21 d (**Table S7**).

### Differentially expressed TFs genes involved in lipid biosynthesis

**Figure 6:**
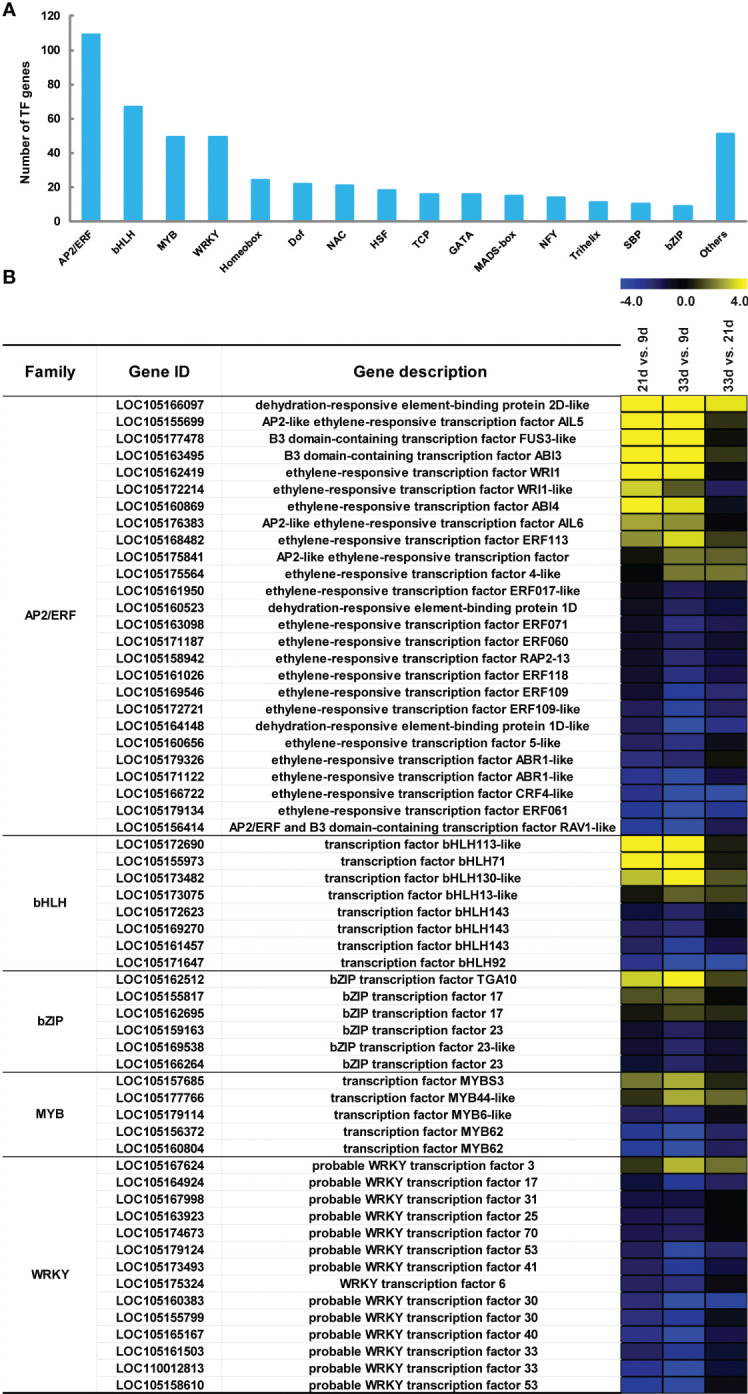
Differential expression of transcription factors (TFs) related to lipid biosynthesis. **(A)** The top 15 differentially expressed TF families. **(B)** Expression changes of the major TF genes with FPKM>1000 at either time point of seed development. The blue and yellow denote down-regulation and up-regulation, respectively.

To obtain a comprehensive understanding of the transcriptional regulation of genes involved in sesame seed development, the differentially expressed TFs related to lipid biosynthesis were identified using Plant TFDB and Pfam databases (**Figure 6A**; **Table S8**). In this study, a total of 492 TF genes belonging to 33 TF families were identified and the top 15 TF families are shown in **Figure 6A**. Of these, the AP2/ERF family had the largest number of members (109, 22%), followed by bHLH (67, 14%), MYB (49, 10%), and WRKY (49, 10%; **Figure 6A**). Our findings also showed that there were five TF family members with high expression levels (FPKM>1000) at least at the early, mid, or late stages of seed development (**Table S8**). Notably, there were a total of 11 AP2/ERF members, including two WRI1 genes, that showed higher expression levels at 21 or 33 d, and their expression pattern was quite similar to many genes related to lipid biosynthesis (**Figure 6B**). Moreover, three MYB and 13 WRKY genes showed higher transcript abundance at 9 d, while lower transcript levels were observed in developing sesame seed at 21 and 33 d (**Figure 6B**).

## Discussion

As oils are an important source of high-density energy and a source of essential FAs in oilseed crops, they are mainly stored in seed. In comparison to other oilseed crops, sesame seed has a relatively high oil content. Lipid accumulation during sesame seed development is a complex process that requires further investigation to uncover the underlying mechanisms (Wang et al., 2014). This study integrated lipidomic and transcriptomic analyses of developing sesame seed to uncover the overall lipid biosynthesis process and several important biosynthesis pathways and candidate genes involved in the lipid composition and content of sesame seed.

### Characteristics of lipids accumulation in developing sesame seed

Oil seed plants accumulate stored lipids primarily through FA synthesis in plastids and TAG synthesis in their ERs through a series of enzyme-catalyzed reactions (Harwood, 1996; Bates et al., 2013). When we measured sesame seed FA composition and content during seed development by GC-MS, we found significant differences in FA content. In line with previous findings in *Brassica napus*, *Glycine max* and *Camellia oleifera*, FA accumulation was slower at the early stage and quicker at the middle and late stages (Woodfield et al., 2017; Lin et al., 2018; Woodfield et al., 2018; Yang et al., 2019). For instance, palmitic acid, stearic acid, oleic acid, and linoleic acid in the current study were the main FAs in developing sesame seed, and their critical period for rapid accumulation was also in the middle and later period of storage. Oleic and linoleic acids are unsaturated FAs and two of the most important components of sesame seed oil, and our findings also show that oleic and linoleic acids are the most abundant at 21d and 33d which determine the quality of the seed oil. The observed alterations in FAs content indicate that certain pivotal genes underwent regulation of expression during sesame seed development. Using the LC-MS method, we found that the lipids DAG and TAG displayed a similar pattern as the main FA accumulation. Remarkably, the accumulation of total PAs was rapidly increasing aftert 21 days, and their proportions remained relatively steady at the late stage which was significantly higher than all other lipids (**Figure 2**). In TAG assembly pathways, the main molecular species of PA were the immediate precursors to DAG, and then DGAT catalyzes the conjugation of a fatty acyl-CoA to DAG, to form TAG. Therefore, the rapid accumulation of DAG and TAG at the late maturation stage of sesame seed development requires substantial amounts of PAs, which suggests PAs have an important role during the accumulation of oil in sesame. The findings suggest that the significant alterations in lipids accumulation are controlled by specific pathways, and the DEGs identified in this study hold potential for identifying regulators of lipid biosynthesis.

### FA biosynthesis and TAG assembly

Although there has been extensive research on the biochemistry of lipid biosynthesis in a wide range of plants, the biosynthesis, transport, and storage of lipids constitute complex processes associated with lipid accumulation and its related pathways are not completely understood (Bates et al., 2013; Chen et al., 2015a; Bates, 2016; He and Ding, 2020). In sesame, it is very evident that our understanding of lipid biosynthesis and accumulation, and particularly its regulation, is incomplete following the discovery of new genes involved in oil production and quality (Wei et al., 2015). In this study, several DEGs associated with FA biosynthesis and TAG assembly pathways were identified among developmental stages, including *ACCase*, *MCAT*, *FAS*, *FAD2*, *FAD3*, *GPAT*, and *DGAT*, which was similar to the findings from a previous study on sesame (Wang et al., 2014). There was, however, a significant difference between these DEGs in terms of their expression patterns during seed development (**Table S6**). For example, the ACCase enzyme is responsible for the first committed step of the FA *de novo* synthesis pathway and overexpression of ACCase genes would lead to an increase in the oil content of plants (Klaus et al., 2004; Chen et al., 2019; Chaturvedi et al., 2021). In addition, during the last and highly important step in TAG biosynthesis, DGAT plays a crucial role in oil accumulation (Weselake et al., 2008; Banas et al., 2013). In a previous study, the DGAT gene was found to be highly expressed in seed and its overexpression in *Arabidopsis* seed increased TAG production (Chen et al., 2016). It is worth noting that several *ACCase* genes showed the highest expression level after 21 d of sesame seed development, whereas two *DGAT* genes were highly expressed at 33 d (**Table S6**). Additionally, FAD2 synthesizes C18:2 from C18:1 and FAD3 converts C18:2 into C18:3 in the ER (Pham et al., 2012; Dar et al., 2017). Previous studies have shown that the knockout of *FAD2* in soybean (Do et al., 2019) and cotton (Chen et al., 2021) and silencing of *FAD2* in flax (Chen et al., 2015b), oilseed rape (Peng et al., 2010), and other plants can dramatically increase C18:1 content. The transcripts of *FAD2* and *FAD3* genes were identified to be significantly decreased at 21 or 33 d, suggesting that 18:2 and C18:3 accumulation may be dependent on the regulation of *FAD2* and *FAD3* expression levels during mid- and late-stage seed development in sesame. These results indicate that genes involved in FA biosynthesis and TAG assembly were important for controlling sesame seed oil content and FA composition, which should be investigated in future research.

### Carbohydrate metabolism

Carbohydrate metabolism pathways, including carbon metabolism, glycolysis/gluconeogenesis, pentose phosphate pathway, and citrate cycle pathway, could provide carbon sources, ATP, and NADPH for lipid biosynthesis, thus indicating that lipid biosynthesis is highly dependent on carbohydrate metabolism (Perry and Harwood, 1994; Hutchings et al., 2005; Furumoto et al., 2011). It was previously demonstrated that 14C-labeled pyruvate can be converted into FAs in isolated plastids from *B. napus* embryos, which suggested that FA biosynthesis could be carried out directly with pyruvate (Eastmond and Rawsthorne, 2000). Several genes encoding important enzymes for the regulation of carbohydrate metabolism have been found to regulate seed oil content, especially *G3PDH*, *PEPCase*, and *PDHC* genes (Yui et al., 2003; Vigeolas et al., 2007; Xu et al., 2016) For instance, *G3PDH* overexpression in rapeseed increased the oil content of transgenic rapeseed to 40% (Vigeolas et al., 2007), while silencing *PEPCase* in cotton by RNAi decreased oil content (Xu et al., 2016), compared to wild type plants. Interestingly, the transcriptomic results in our present work suggest that many DEGs are involved in carbohydrate metabolism pathways, especially *G3PDH*, *PDHC*, *PEPCase*, and *PK* genes, which were found to be significantly differentially expressed during sesame seed development (**Table S7**). Clarifying the regulation of carbohydrate metabolism might contribute more carbon sources to lipid biosynthesis and provide an important reference to increasing the oil content in oilseed crops through breeding. Therefore, our findings reveal that these putative genes may play important roles in the process of lipid biosynthesis and accumulation during sesame seed development. More studies are needed to verify the gene functions and explore their associated regulatory mechanisms.

### TFs involved in the regulation of lipid biosynthesis and accumulation

Plant seeds accumulate lipids through a complex and well-organized biological process involving carbohydrate metabolism, FA biosynthesis, and TAG assembly pathways, and the up-regulation of multiple related genes is likely required for greater lipid accumulation. Researchers have discovered that several important TFs are involved in the regulation of lipid biosynthesis and accumulation in recent years (Tan et al., 2011; Liu et al., 2014; Chen et al., 2020). Changing the expression level of *BnGRF2* (Liu et al., 2012), *WRI1* (Chen et al., 2020), *MYB73* (Liu et al., 2014), *GmbZIP123* (Song et al., 2013), and *DOF1/4* (Wang et al., 2007) in *Arabidopsis* and *BnLEC1* (Tan et al., 2011), NFYA (Lu et al., 2016), and *GmWRI1s* (Chen et al., 2020; Guo et al., 2020) in oilseed crops can significantly improve the oil content in plant seed. Through genetic engineering, oil synthesis-related TFs may thus represent promising targets for enhancing oil yields in oilseed crops. In this study, a systematic search for sesame TFs involved in lipid accumulation was conducted by identifying genes expressed more strongly during rapid lipid synthesis. Our work showed that a total of 33 TF families were involved in lipid accumulation and five TF family members showed high expression levels (FPKM > 1000 at least at one time point) during seed development in sesame, including AP2/ERF, bHLH, bZIP, MYB, and 13 WRKY TF family. Many studies have demonstrated that *WRI1* encodes an AP2/ERF TF, which regulates carbon partitioning into oils and proteins and controls the expression of many genes essential for FA synthesis (Sakuma et al., 2002; Baud et al., 2007; Kong and Ma, 2018; Kong and Ma, 2018; Lu et al., 2021). Remarkably, there were several AP2/ERF TF members, especially WRI1 and WRI1-like genes in sesame that showed higher levels of expression at 21 or 33 d, with expression patterns similar to many genes involved in lipid biosynthesis, suggesting their important position in the process of sesame seed oil accumulation. Additionally, in addition to AP2/ERFTF expression, we proposed that the high transcript levels of other TFs suggest these have a role in oil accumulation in sesame seed as well.

## Conclusions

In the present work, we describe lipidomic and transcriptomic analyses of major lipids and genes related to lipid biosynthesis and its accumulation in the oil-rich crop, *Sesamum indicum* L. A targeted lipidomic analysis showed a variety of molecular species in each class of lipid, and the critical period for the rapid accumulation of most lipids was found to be during the middle and later periods of storage in developing sesame seed. Furthermore, the transcriptomic analysis showed that a large number of DEGs were enriched in lipid biosynthesis and metabolic, carbohydrate, and energy metabolism pathways, and a number of candidate genes (such as *ACCase*, *FAD2*, and *DGAT*) were identified as having an impact on sesame seed oil content and FA composition. In conclusion, these results will improve our understanding of the molecular basis of lipid biosynthesis and accumulation, which provides good theoretical guidance and technical support for the molecular improvement of sesame.

## Data availability statement

The datasets presented in this study can be found in online repositories. The names of the repository/repositories and accession number(s) can be found below: https://ngdc.cncb.ac.cn/gsa/browse/CRA008917, Genome Sequence Archive.

## Author contributions

YZ, JZ and XZ designed and conceived the study. HG, XC, CG, NL and YP performed the experiments and the data analysis. YZ, JZ and XZ wrote and edited the manuscript. All authors contributed to the article and approved the submitted version.
