# Supplementary material for: Integrated lipidomic and transcriptomic analyses reveal the mechanism of lipid biosynthesis and accumulation during seed development in sesame
Source: Front Plant Sci. 2023 Jun 22;14:1211040. doi: 10.3389/fpls.2023.1211040 (PMC10325577; doi:10.3389/fpls.2023.1211040)
Supplement: Supplementary file 1 [file DataSheet_1.doc]

**Supplementary information**

**Table S1. Gene primers for qRT-PCR.**

**Table S2. Fatty acid composition and content in developing sesame seed.**

**Table S3. The composition and content of differentially accumulated lipids in developing sesame seed.**

**Table S4. Summary of RNA-seq data and mapping quality.**

**Table S5. Significantly enriched KEGG pathways of the DEGs in developing sesame seed.**

**Table S6. Expression changes of the genes involved in FA biosynthesis and TAG assembly in developing sesame seed.**

**Table S7. Expression pattern of important genes related to carbon metabolism.**

**Table S8.** **Differentially expressed transcription factors related to lipid biosynthesis.**

**Figure S1.** **Principal component analysis (PCA) of lipid profiles.** Quality control samples (QC) were prepared by mixing equal quantities of 9, 21, and 33 d samples.

**Figure S2. Correlation matrix between nine RNA-Seq samples.**

**Figure S3. qRT-PCR and RNA-Seq correlation analysis using log_2_fold change for selected genes.**
