# Supplementary figures and images for: Integrated lipidomic and transcriptomic analyses reveal the mechanism of lipid biosynthesis and accumulation during seed development in sesame
Source: Front Plant Sci. 2023 Jun 22;14:1211040. doi: 10.3389/fpls.2023.1211040 (PMC10325577; doi:10.3389/fpls.2023.1211040)

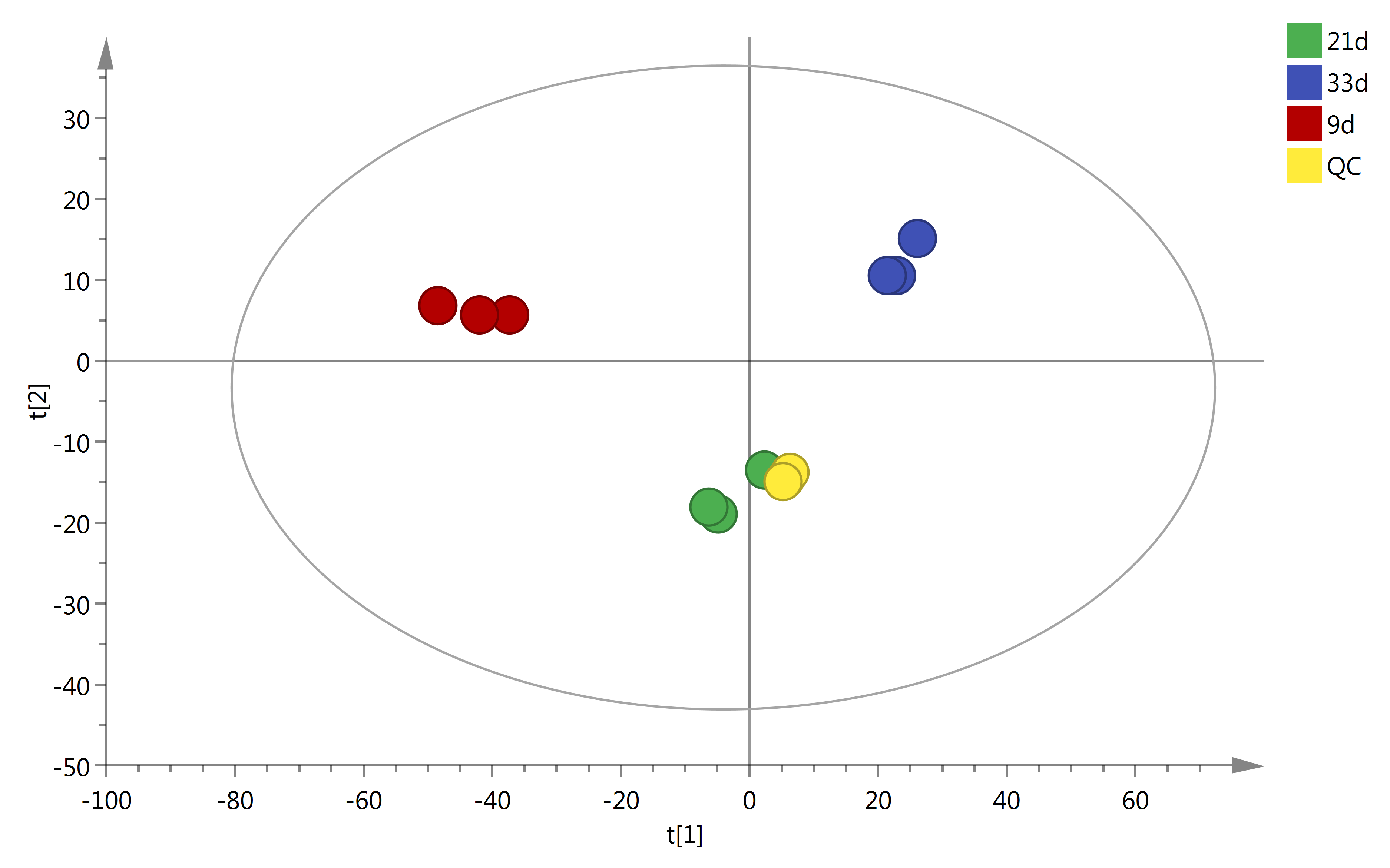

Supplement: Supplementary file 2 [file Image_1.png]

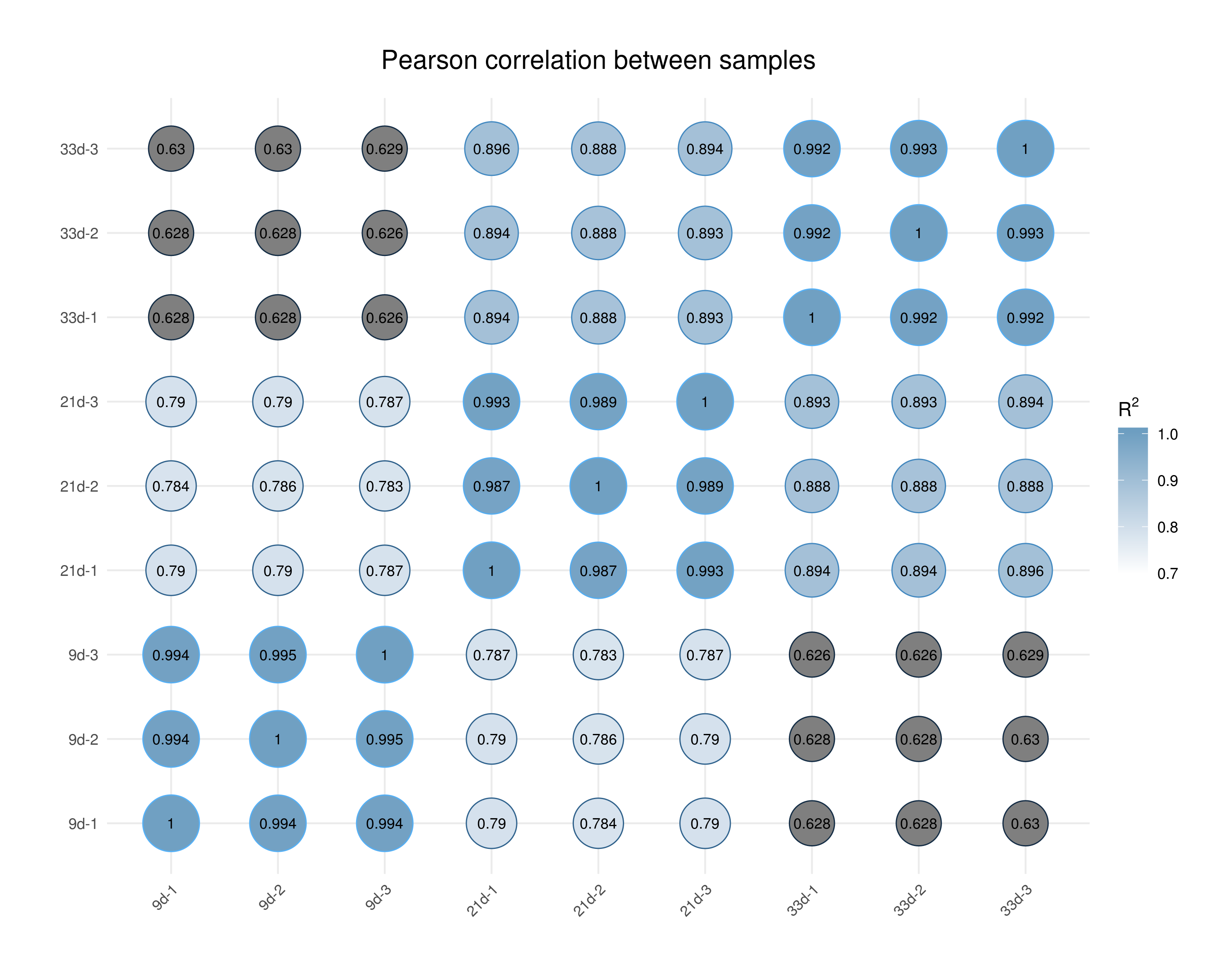

Supplement: Supplementary file 3 [file Image_2.png]

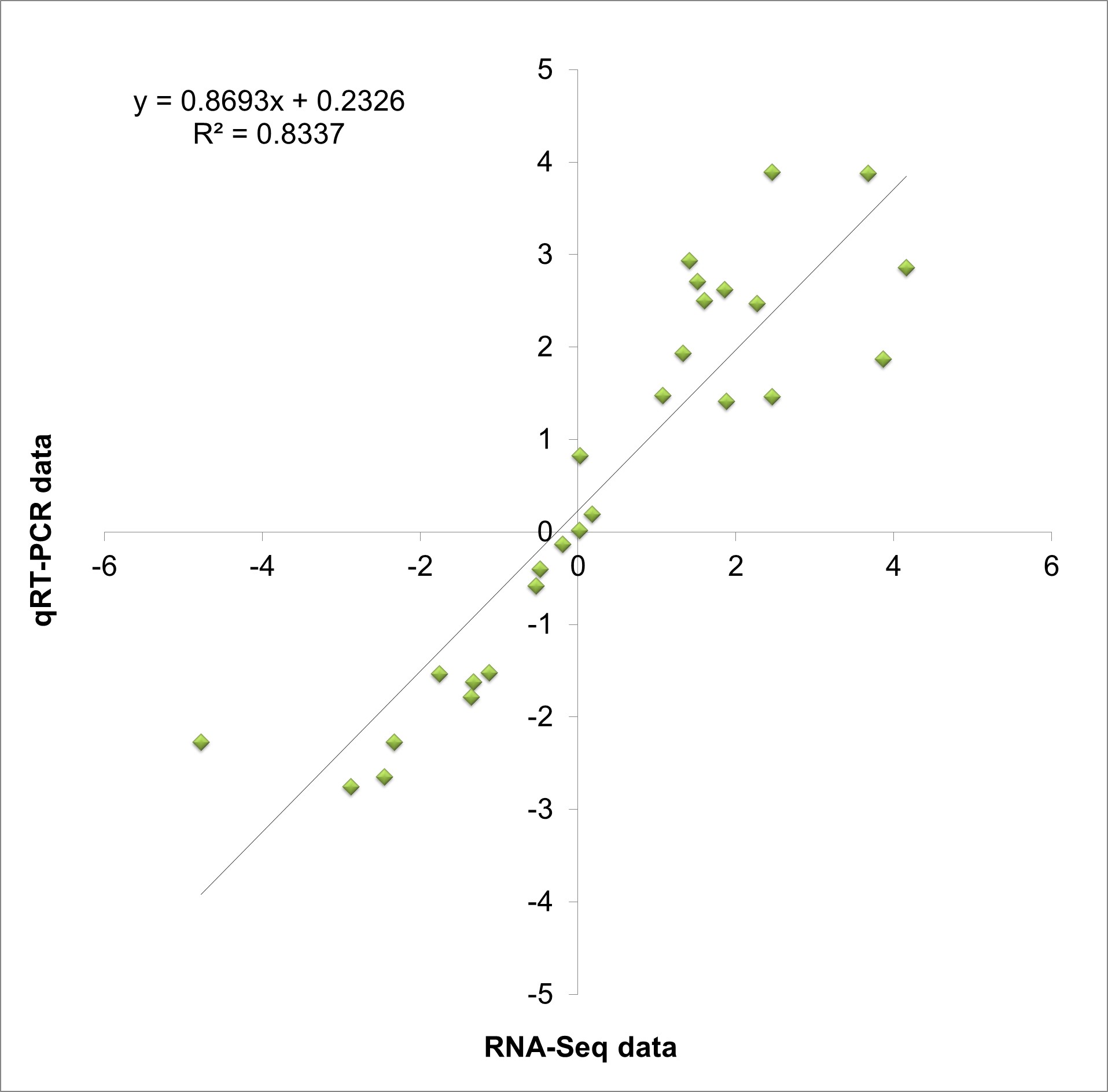

Supplement: Supplementary file 4 [file Image_3.jpeg]
